# Supplementary material for: Single-cell spatial architectures associated with clinical outcome in head and neck squamous cell carcinoma
Source: NPJ Precis Oncol. 2022 Feb 25;6:10. doi: 10.1038/s41698-022-00253-z (PMC8881577; doi:10.1038/s41698-022-00253-z)
Supplement: Supplementary file 1 — Supplementary Figures 1-3 [file 41698_2022_253_MOESM1_ESM.pdf]

a

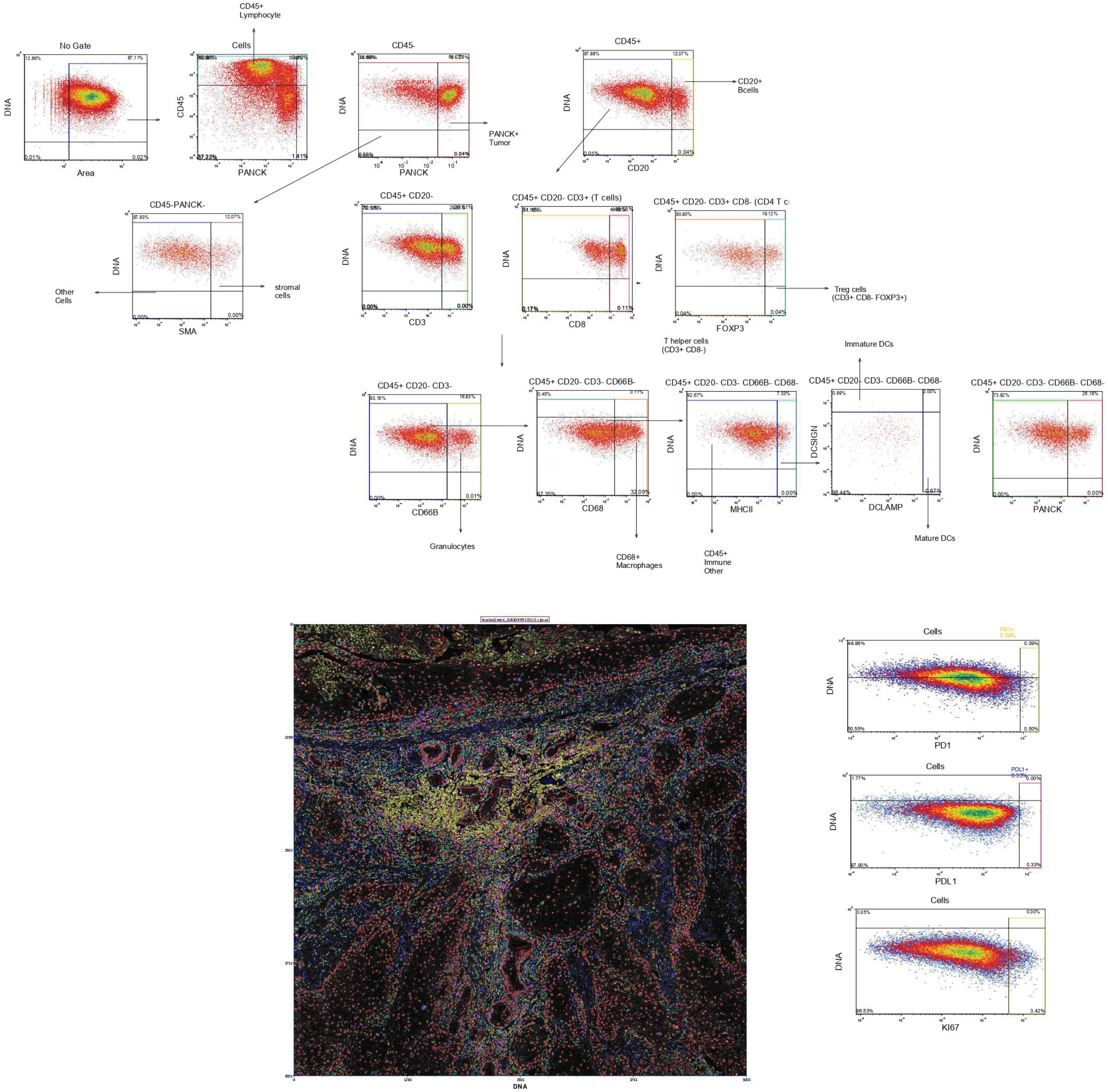

Supplementary Figure 1. a, Gating template used to determine cell phenotypes and classification of ten populations using image gating cytometry in FCS Image Cytometry 7 RUO. Scatter plots show single cell mean intensity distributions rescaled from 0-1 on a log10 scale with gated populations in colored boxes. Image plot shows a semi-transparent pseudo-colored mask of each cell object colored by the classified population overlaid on signal extracted nuclei image. The markers used for identification of cell phenotypes are shown in Table 1.

b

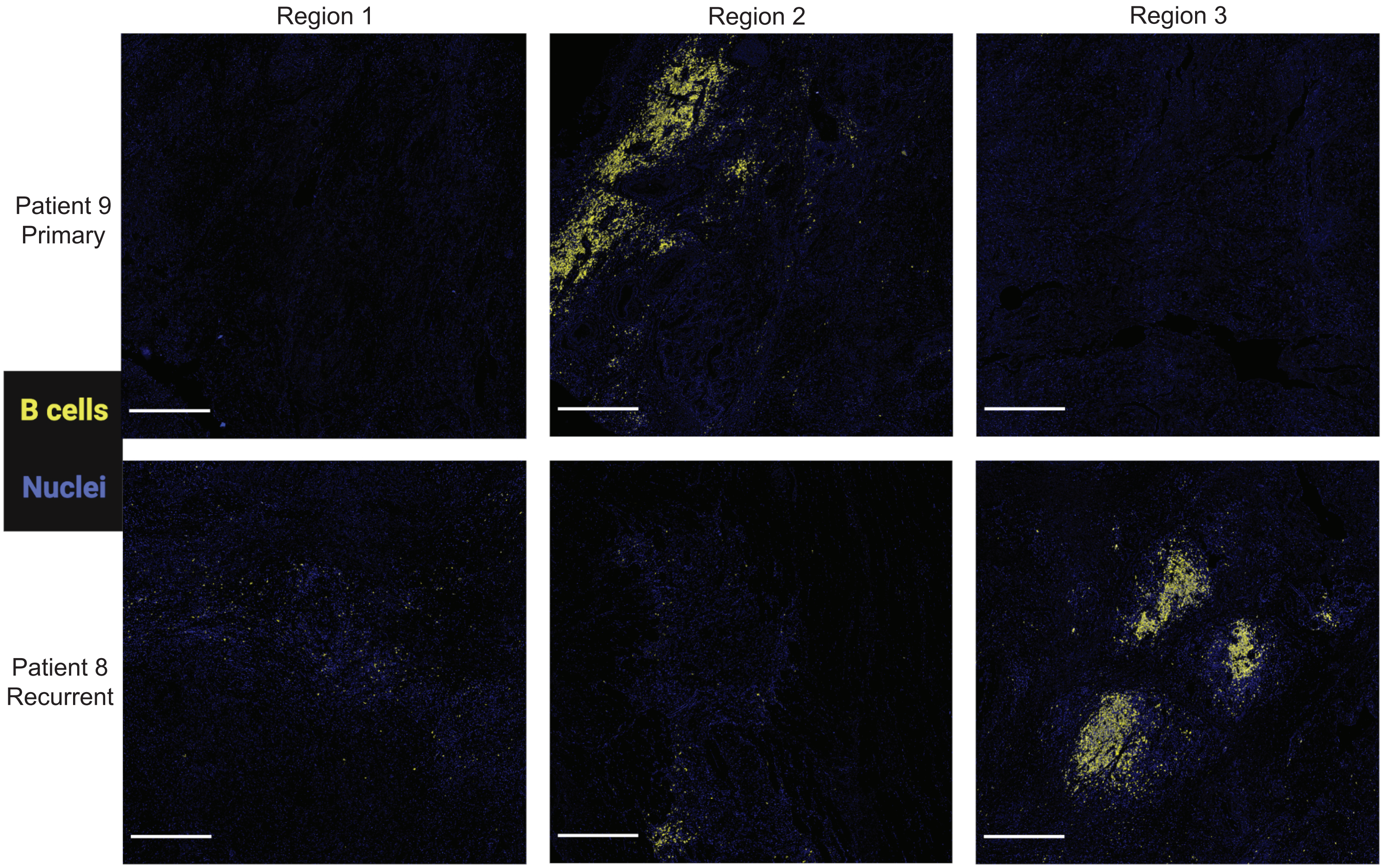

c

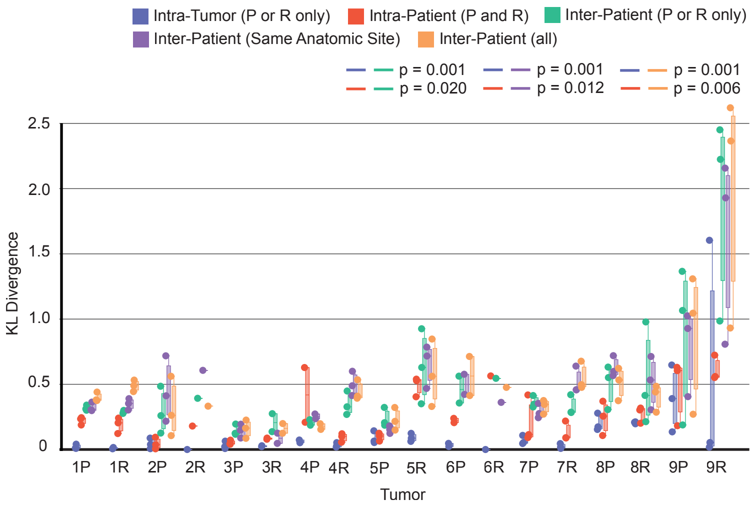

Supplementary Figure 1. b, mIHC images of six tumor regions selected from two different tumor resections (patient 9 primary and patient 8 recurrent). Blue pseudo-color indicates all cell nuclei present in the region (Hematoxylin^{+}) and yellow indicates B cells. In both tumors, two regions have low densities of B cells, whereas one region has a high density and spatial concentration of B cells, despite the three regions being sampled from the same tumor. **c**, Box plot of the Kullback-Leibler divergences from a single tumor region's immune cell distribution compared to: the tumor's average immune cell distribution [Intra-Tumor (P or R only)], the patient's average immune cell distribution [Intra-Patient (P and R)], the cohort's average immune cell distribution across tumors of the same timepoint [Inter-Patient (P or R only)], the cohort's average immune cell distribution across tumors of the same anatomic site [Inter-Patient (Same Anatomic Site)], the cohort's average immune cell distribution across all tumors from all patients [Inter-Patient (all)]. P-values calculated using a one-way ANOVA multi-group significance test followed by a Tukey honestly significant difference post-hoc test.

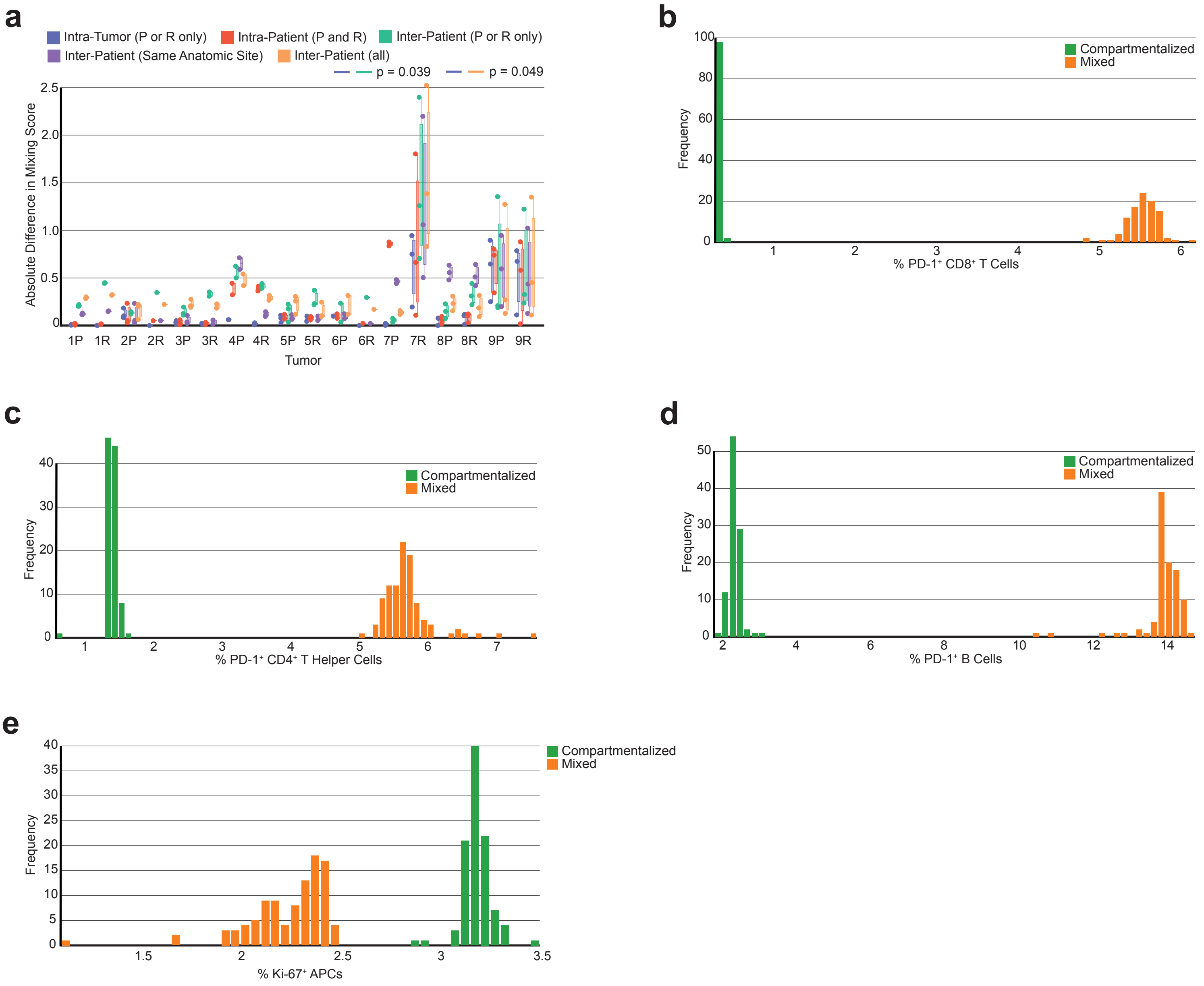

Supplementary Figure 2. a, Box plot of the absolute difference from a single tumor region's mixing score compared to: the tumor's average mixing score [Intra-Tumor (P or R only)], the patient's average mixing score [Intra-Patient (P and R)], the cohort's average mixing score across tumors of the same timepoint [Inter-Patient (P or R only)], the cohort's average mixing score across tumors of the same anatomic site [Inter-Patient (Same Anatomic Site)], the cohort's average mixing score across all tumors from all patients [Inter-Patient (all)]. P-value calculated using a one-way ANOVA multi-group significance test followed by a Tukey honestly significant difference post-hoc test. **b**, Histogram showing the results of a bootstrapping analysis on the percentage of $CD8^+$ T cells expressing PD-1. Bars are colored by spatial architecture. **c**, Histogram showing the results of a bootstrapping analysis on the percentage of $CD4^+$ T helper cells expressing PD-1. Bars are colored by spatial architecture. **d**, Histogram showing the results of a bootstrapping analysis on the percentage of B cells expressing PD-1. Bars are colored by spatial architecture. **e**, Histogram showing the results of a bootstrapping analysis on the percentage of APCs expressing Ki-67. Bars are colored by spatial architecture.

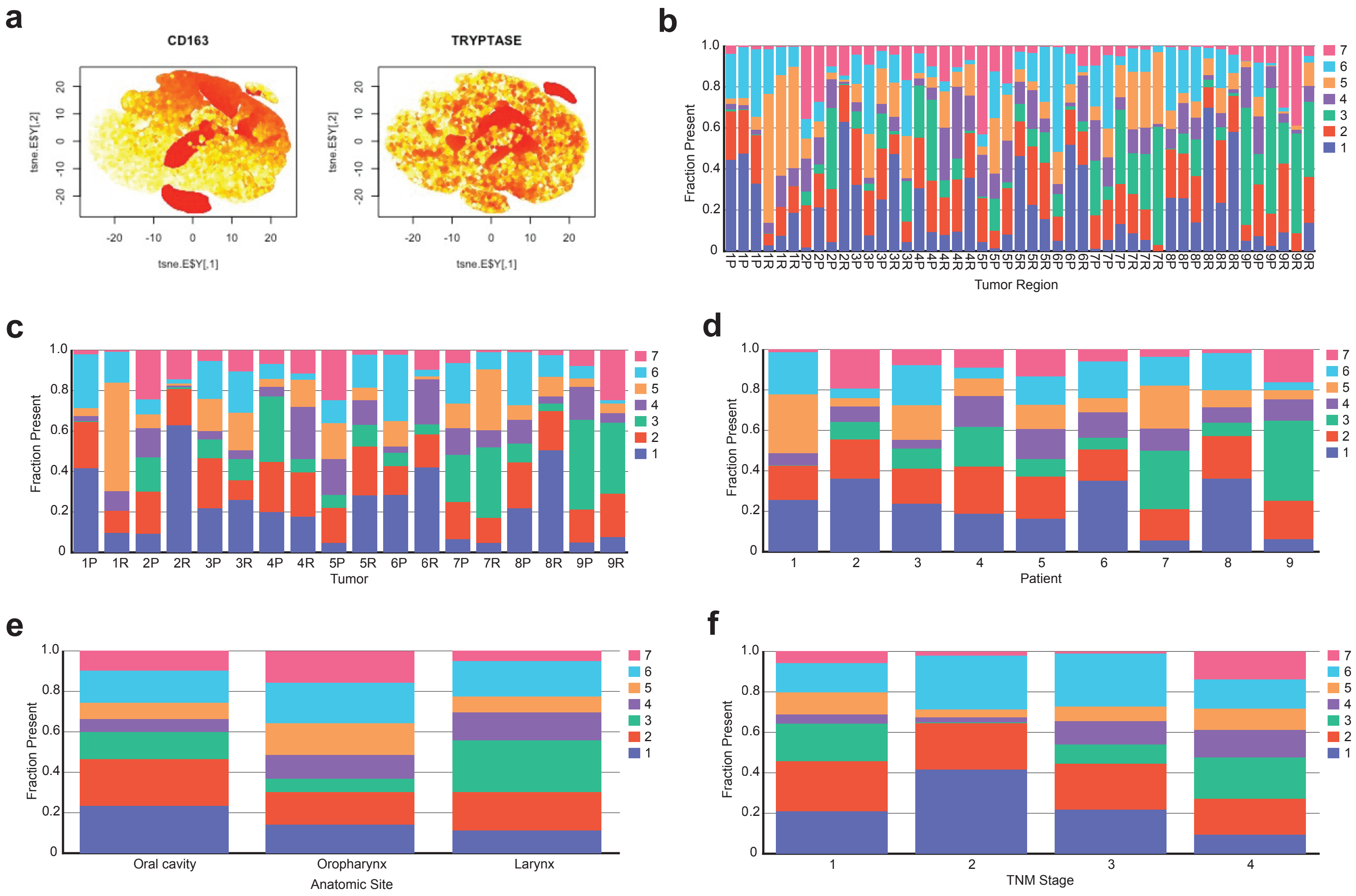

Supplementary Figure 3. a, t-SNE representation of single cells from the 'Other Immune' class colored by heatmap of mean intensity for CD163 and tryptase (mast cells) show distinct populations of cells expressing high levels of CD163 and tryptase, indicating these cell types are likely present in the 'Other Immune' class. **b**, Stacked bar chart showing the proportion (out of 1.0) of αSMA^+ cell neighborhood clusters present in each tumor region (n=47). **c**, Stacked bar chart showing the average proportion (out of 1.0) of αSMA^+ cell neighborhood clusters present in each tumor (n=18). **d**, Stacked bar chart showing the proportion (out of 1.0) of αSMA^+ cell neighborhood clusters present in each patient's primary and recurrent tumors averaged (n=9). **e**, Stacked bar chart showing the average proportion (out of 1.0) of αSMA^+ cell neighborhood clusters present in tumors collected from the three anatomic sites. **f**, Stacked bar chart showing the average proportion (out of 1.0) of αSMA^+ cell neighborhood clusters present in the primary tumors by their TNM stage.
