# Supplementary material for: Single-cell spatial architectures associated with clinical outcome in head and neck squamous cell carcinoma
Source: NPJ Precis Oncol. 2022 Feb 25;6:10. doi: 10.1038/s41698-022-00253-z (PMC8881577; doi:10.1038/s41698-022-00253-z)
Supplement: Supplementary file 2 — REPORTING SUMMARY [file 41698_2022_253_MOESM2_ESM.pdf]

nature portfolio

Corresponding author(s): Jeremy Goecks

Last updated by author(s): Dec 9, 2021

Reporting Summary

Nature Portfolio wishes to improve the reproducibility of the work that we publish. This form provides structure for consistency and transparency in reporting. For further information on Nature Portfolio policies, see our Editorial Policies and the Editorial Policy Checklist.

Statistics

For all statistical analyses, confirm that the following items are present in the figure legend, table legend, main text, or Methods section.

n/a	Confirmed	
☐	☒	The exact sample size (*n*) for each experimental group/condition, given as a discrete number and unit of measurement
☐	☒	A statement on whether measurements were taken from distinct samples or whether the same sample was measured repeatedly
☐	☒	The statistical test(s) used AND whether they are one- or two-sided<br>*Only common tests should be described solely by name; describe more complex techniques in the Methods section.*
☐	☒	A description of all covariates tested
☐	☒	A description of any assumptions or corrections, such as tests of normality and adjustment for multiple comparisons
☐	☒	A full description of the statistical parameters including central tendency (e.g. means) or other basic estimates (e.g. regression coefficient) AND variation (e.g. standard deviation) or associated estimates of uncertainty (e.g. confidence intervals)
☐	☒	For null hypothesis testing, the test statistic (e.g. *F*, *t*, *r*) with confidence intervals, effect sizes, degrees of freedom and *P* value noted<br>*Give P values as exact values whenever suitable.*
☒	☐	For Bayesian analysis, information on the choice of priors and Markov chain Monte Carlo settings
☒	☐	For hierarchical and complex designs, identification of the appropriate level for tests and full reporting of outcomes
☐	☒	Estimates of effect sizes (e.g. Cohen's *d*, Pearson's *r*), indicating how they were calculated

Our web collection on statistics for biologists contains articles on many of the points above.

Software and code

Policy information about availability of computer code

Data collection	Image processing was performed using MATLAB, CellProfiler Version 2.2.0, and FCS Express 7 Image Cytometry RUO.
Data analysis	All data analyses were performed using Python software, version 3.6.5. The code created to produce the results of this study is available at https://github.com/kblise/HNSCC_mIHC_paper.

For manuscripts utilizing custom algorithms or software that are central to the research but not yet described in published literature, software must be made available to editors and reviewers. We strongly encourage code deposition in a community repository (e.g. GitHub). See the Nature Portfolio guidelines for submitting code & software for further information.

Data

Policy information about availability of data

All manuscripts must include a data availability statement. This statement should provide the following information, where applicable:

- Accession codes, unique identifiers, or web links for publicly available datasets
- A description of any restrictions on data availability
- For clinical datasets or third party data, please ensure that the statement adheres to our policy

All of the data produced by our mIHC computational image processing pipeline, including protein abundance, cell phenotype, and cell location information saved in the form of a matrix, in addition to survival data, is available for download on Zenodo at https://doi.org/10.5281/zenodo.5540356.

Field-specific reporting

Please select the one below that is the best fit for your research. If you are not sure, read the appropriate sections before making your selection.

☒ Life sciences ☐ Behavioural & social sciences ☐ Ecological, evolutionary & environmental sciences

For a reference copy of the document with all sections, see nature.com/documents/nr-reporting-summary-flat.pdf

Life sciences study design

All studies must disclose on these points even when the disclosure is negative.

Sample size	Original cohort enrolled 20 patients with matched primary and recurrent tumor resections. Following mIHC staining, tissue loss occurred for tumor samples in 11 patients, resulting in a final cohort size of 9 patients (18 tumors). Each tumor had 1-3 regions assayed with mIHC, resulting in 47 tumor regions.
Data exclusions	Patients whose primary or recurrent tumor experienced tissue loss following mIHC staining were removed from downstream analyses (n=11).
Replication	None of the findings were replicated due to human tissue data availability.
Randomization	Not relevant to study, as all samples were analyzed together in bulk.
Blinding	Blinding was not relevant to study, as all samples were analyzed together in bulk.

Reporting for specific materials, systems and methods

We require information from authors about some types of materials, experimental systems and methods used in many studies. Here, indicate whether each material, system or method listed is relevant to your study. If you are not sure if a list item applies to your research, read the appropriate section before selecting a response.

Materials & experimental systems

n/a	Involved in the study	
☐	☒	Antibodies
☒	☐	Eukaryotic cell lines
☒	☐	Palaeontology and archaeology
☒	☐	Animals and other organisms
☐	☒	Human research participants
☒	☐	Clinical data
☒	☐	Dual use research of concern

Methods

n/a	Involved in the study	
☒	☐	ChIP-seq
☒	☐	Flow cytometry
☒	☐	MRI-based neuroimaging

Antibodies

Antibodies used	See Banik et al., 2020 for complete list of antibodies used, including vendor, product/clone number, etc.
Validation	See Banik et al., 2020 for description of antibody validation.

Human research participants

Policy information about studies involving human research participants

Population characteristics	Briefly, patients included 7 females and 2 males, all presenting with HNSCC. See Table 1 for more details, including HPV status, alcohol use, tobacco use, and treatment regimen.
Recruitment	Tissue was obtained from the Oregon Health & Science University Knight Biolibrary.
Ethics oversight	All studies involving human tissue were approved by IRB (protocol #809 and #3609), and written informed consent was obtained.

Note that full information on the approval of the study protocol must also be provided in the manuscript.
